# Benefit–risk balance of native vitamin D supplementation in chronic hemodialysis: what can we learn from the major clinical trials and international guidelines?

**DOI:** 10.1080/0886022X.2019.1632719

**Published:** 2019-07-03

**Authors:** Yassamine Bentata

**Affiliations:** a Nephrology Unit, University Hospital Mohammed VI, Oujda, Morocco;; b Laboratory of Epidemiology, Clinical Research and Public Health, Medical School of Oujda, University Mohammed The First, Oujda, Morocco

**Keywords:** Native vitamin D, chronic hemodialysis, clinical trials, guidelines

## Abstract

For some years, there has been a great renewal of interest in native vitamin D and its major involvement in osseous and non-osseous effects in the organism. Patients in chronic hemodialysis (CHD) constitute a specific population with different physiopathologic characteristics and needs, since morbidity and mortality are strongly correlated with vitamin D insufficiency. Vitamin D supplementation raises very pertinent questions for which we have only partial answers and we lack solid scientific proof to establish certain truths. Thus, we try through this mini-review to analyze the results of the main randomized clinical trials conducted during the last decade, and to discuss international guidelines concerning native vitamin D supplementation in CHD patients. Seven double-blind randomized clinical trials have evaluated native Vitamin D supplementation in CHD patients. These clinical trials began between 2007 and 2013 and studied relatively small samples of patients with an average of 50. All of these trials are important, but do not provide sufficient scientific proof concerning the advantages, consequences, and secondary effects of native vitamin D supplementation in CHD. None of the European, American, English, Asian, Australian, or Canadian recommendations have specified the targets, doses, duration, or the molecule of vitamin D supplementation in the patient on CHD. In 2017, the long-awaited KDIGO recommendations were published and despite the results of clinical trials conducted, the recommendations on native vitamin D supplementation in CHD were very imprecise and sparse, limited to suggesting correction of any state of vitamin D insufficiency or deficiency.

## Introduction

For some years, there has been a great renewal of interest in native vitamin D and its major involvement in osseous and non-osseous effects in the organism, whether in general or specific populations. Native vitamin D insufficiency is strongly present in Chronic Kidney Disease (CKD), widely observed in chronic hemodialysis (CHD), of multifactorial origin, and correlated with high morbidity and mortality [1–3]. Vitamin D is one of the major biochemical parameters in entity mineral and bone disorders (MBD), a concept developed in 2005 and published in 2009 by Kidney Disease Improving Global Outcomes (KDIGO) to describe all abnormalities of phosphocalcic metabolism observed in patients with CKD [4]. Following a better illustration of the role of vitamin D in CKD-MBD, the use of vitamin D supplementation in patients with CKD over the past two decades has been significantly increased, but there is no consensus for vitamin D, particularly in patients with CHD because of the scarcity of clinical trials conducted on this topic. The aim of this mini-review is to briefly present the physiopathologic elements of native vitamin D, analyze the results of the main published clinical trials, and to discuss the recommendations concerning native vitamin D supplementation in CHD.

## Physiopathology of native vitamin D and special features in hemodialysis

Vitamin D_3_ (cholecalciferol) and vitamin D_2_ (ergocalciferol) constitute the two forms at the origin of native vitamin D. More than 90% comes from ultraviolet sun rays of type B (UVB) and less than 10% is of nutritional plant and animal origin. Its half-life is 6 months and it is mainly stored in adipose tissue [5]. As a result, thin persons have a lower storage capacity and may easily become deficient, while obese patients require higher doses of vitamin D to reach their physiologic needs. Vitamin D (D_2_ and D_3_) is transported in the blood and undergoes a first hydroxylation in the liver at the carbon 25 position under the action of D-25 hydroxylase, to produce 25 hydroxyvitamin D (25(OH) D) or calcidiol. Calcidiol undergoes a second hydroxylation in the kidney at the carbon 1 position under the action of D-1,25 hydroxylase to produce 1,25 dihydroxyvitamin D (1,25(OH)_2_D) or calcitriol. There is a pathway of calcitriol inactivation, 24 hydroxylase, the expression of which in the proximal renal tubule is stimulated by Fibroblast Growth Factor 23 (FGF23) and calcitriol. 25(OH)D, precursor of active vitamin D, has a half-life of 15–30 days and is the best marker of the vitamin D status of the organism. Its production is not well-regulated and remains highly dependent on the body's production of vitamin D_2_ and D_3_ under the main action of UVB rays. The more vitamin D is synthesized, the more 25(OH)D is produced. It is very important to specify the three groups of forms of vitamin D supplementation usually used to treat bone disorders of CKD. The first group corresponds to native vitamin D: vitamin D_2_ analogs, vitamin D_3_ analogs, and calcifediol (25(OH)D_3_). The second group corresponds to active analog vitamin D, in the form of nonselective vitamin D-receptor activators, such as calcitriol (first generation) and alfacalcidol 1α OHD_3_ (second generation). The third group corresponds to active analog vitamin D, in the form of selective vitamin D-receptor activators, such as paricalcitol 19-nor-1α (third generation) and alfacalcidol 1α OHD_3_ (second generation) 25 hydroxyvitaminD_2_, and doxercalciferol 1α hydroxyvitaminD_2_. The term selective means that the molecule acts mostly on the parathyroid gland, more so than on intestine and bone, resulting in lower serum calcium and phosphorus blood concentrations. In this review, we will use the term native vitamin D to refer to the two vitamin molecules: vitamin D_3_ (Cholecalciferol) and vitamin D_2_ (Ergocalciferol).

Bone and mineral disorders (BMD) comprise one of the earliest, most common, and most serious complications of CKD whose severity increases with the severity of chronic kidney failure. This group of disorders corresponds to a wide spectrum of disturbances affecting three aspects of phosphocalcic metabolism; biological abnormalities of calcium, phosphorus, parathormone, vitamin D, and FGF23; the morpho-histological aspect of bone (bone volume, bone turnover, bone mineralization) and diffuse calcification of soft tissue, blood vessels, and cardiac valves. The main form encountered is secondary hyperparathyroidism, which is biologically characterized by FGF23 elevation, hypocalcemia, and hyperphosphoremia, vitamin D deficiency, and stimulation of parathyroid hormone secretion [6,7]. In this context, the deficit in active vitamin D is largely related to the decrease in the production of 1α hydroxylase, secondary to renal parenchymal fibrosis and the production of FGF23, but also to the decrease in the production of 25(OH)D.

The definition of 25(OH)D status among patients in CHD has not given rise to any recent consensual revision. At this time, we use that developed by the KDIGO 2009 group and in which vitamin D insufficiency is defined by a vitamin D level between 16 and 30 ng/mL and vitamin D deficiency by a level of <16 ng/mL [4]. A related status of severe deficiency is defined by a vitamin D level of <5 ng/mL. On the one hand, it is important to point out that these thresholds were validated in the general population among healthy persons, in whom thresholds were set based on studying the concentration level of 25(OH)D, from which the value of plasma parathormone (PTH) may increase. This threshold of 30 ng/mL was used for several studies in which the value of plasma PTH was lowered for plasma values of vitamin D > 30 ng/mL in a population of osteoporotic patients being treated with bisphosphonates, a medication that lowers the plasma concentration of PTH, and not in patients with CKD [8–10]. In the latter, variations in vitamin D concentration are closely related to calciotropic and phosphaturic hormones such as PTH and FGF23, calcemia, phosphoremia, medications interfering with phosphocalcic metabolism, as well as to other clinical and biological characteristics of the CHD patient. We use default thresholds for CHD patients close to those retained for the general population without definite scientific proof. On the other hand, the toxicity threshold of vitamin D is also poorly defined in CHD patients. It is very important to specify that resulting complications, especially cardiovascular calcifications, are strongly correlated with high morbi-mortality in CHD. Although the toxicity threshold of vitamin D is 150 ng/mL in the general population, it seems advisable not to exceed the threshold of 80 ng/mL in CHD patients. The rationale is to avoid the appearance not only of complications specific to CKD, such as valvular, cardiac, and vascular calcifications following increased intestinal absorption of phosphorus and calcium, but also other pathologies not specific to CKD, particularly neoplasia [11,12]. These proposed thresholds are not very precise because they are not really established, but are rather ‘desirable’ or ‘recommended’ values within acceptable ranges, in view of achieving optimal control of phosphocalcic and osseous metabolism, and of the cardiovascular, immune, and inflammatory systems. Vitamin D insufficiency varies according to numerous parameters, the stage of CKD (greater deficiency in case of ESRD), factors linked to CKD, and other factors independent of CKD. Factors nonspecific to CKD and the most frequently found, comprise dietary restrictions with interdiction of numerous vitamin D-enriched products, lack of appetite, insufficient sun exposure, diminution of cutaneous synthesis, the season of the blood sample, advanced age, female gender, ethnicity, obesity, diabetes, pigmented skins, and socio-cultural habits [13–15].

Other specific and molecular factors are also involved in vitamin D deficiency in CHD, such as resistance and/or decreased sensitivity of the tissue receptors of vitamin D, decreased hepatic synthesis of CYP24A1, decreased tubular absorption of 25(OH)D due to the decrease of megaline, decreased activity of 1α hydroxylase secondary to uremia and acidosis, and increased activity of 24–25(OH)_2_D [16–19]. Secondary hyperparathyroidism, decrease of bone mineral density, muscular weakness, falls and fractures, metabolic syndrome and obesity, insulin resistance, left ventricular hypertrophy, atherosclerosis, vascular and valvular calcifications, alteration of mental faculties, and progression of kidney disease from the pre-dialysis stage to the dialysis stage, are the main consequences of vitamin D deficiency in CHD patients [20–24]. The association of mortality and vitamin D has been the subject of many studies which converge on a strong association between mortality and vitamin D deficiency in CKD, including in the early stages [25,26]. Pilz et al. noted in their meta-analysis that the decrease in vitamin D plasma concentration of 10 ng/mL was associated with a 14% increase of mortality risk and the decrease of 25(OH)D and of 1.25(OH)_2_D was associated with mortality among patients on hemodialysis. In the French cohort of Jean et al., the risk of mortality increased up to 30% when vitamin D plasma concentrations were less than 18 ng/mL [27].

## Randomized clinical trials

Is substitution for vitamin D deficiency required in CHD patients? The answer is increasingly obvious in view of the central role of vitamin D in MBDs, the prevention of fractures and the optimization of extra-osseous effects of vitamin D, especially cardiovascular. While substitution in vitamin D deficiency is no longer a matter of debate, doses, methods of administration, and threshold definitions are still far from being well-established.

Seven double-blind randomized clinical trials have evaluated native vitamin D supplementation in adult CHD patients. These clinical trials began between 2007 and 2012 and studied relatively small numbers of patients with an average of 50 patients for the two groups (vitamin D versus placebo). The results of the main double-blind clinical trials over the last decade are reported in Table 1. The choice of clinical trials mentioned in Table 1 is based on the fact that these are the only randomized controlled double-blind (placebo versus native vitamin D) trials published in the literature during the last two decades concerning native vitamin D supplementation in the CHD patient.

**Table 1.** Results of the main randomized controlled trial Double-Blind (RCT- DB) published in the last decade in adult chronic hemodialysis patients (>18 years).

Randomized Trial	Bhan et al. [28]	Armas et al. [29]	Delanaye et al. [30]	Marckmann et al. [31]	Wasse et al. [32]	Hewitt et al. [33]	Massart et al. [34]
Study period	2009–2013	2007–2010	2009–2010	2009–2009	2009–2010	2011–2011	2008–2009
RCT-double blind	Placebo versus vitD	Placebo versus vitD	Placebo versus vitD	Placebo versus vitD	Placebo versus vitD	Placebo versus vitD	Placebo versus vitD
Mono or multicenter	Multicenter	Multicenter	Multicenter	Monocenter	Monocenter	Monocenter	Multicenter
Number of patients				All patients *N* = 52			
Total	*N* = 105	*N* = 42	*N* = 30	Hemodialysis group *N* = 27	*N* = 52	*N* = 60	*N* = 55
VitD group	Weekly arm vitD = 36	VitD = 20	VitD = 16	VitD G = 13	VitD = 25	VitD= 30	VitD G = 26
Placebo group	Monthly arm vitD = 33 Placebo arm = 36	Placebo = 22	Placebo = 14	Placebo G =14	Placebo = 27	Placebo = 30	Placebo G = 29
Mean age, years VitD group Placebo group	Weekly vitD: 53 ±17 Monthly vitD:58 ±16 Placebo: 59 ±17	VitD: 57.6 Placebo: 54.3	VitD: 75 ± 9 Placebo: 73 ± 12	For all patients VitD: 71 Placebo: 68	VitD: 49 ± 13 Placebo: 52 ± 14	VitD: 60 Placebo: 67	VitD: 62 ± 12 Placebo: 66 ±12
Sex, Male, %	Weekly vitD: 69.4 Monthly vitD: 84.9 Placebo: 80.6	VitD: 70 Placebo: 72	VitD: 75 Placebo: 64	For all patients VitD: 73 Placebo: 76	VitD: 60 Placebo: 63	VitD: 53 Placebo: 43	VitD: 69 Placebo: 55
Ethnicity White versus Black, %	Weekly: 63.6 versus 36.1 Monthly: 63.6 versus 27.3 Placebo: 61.1 versus 25	VitD: 50 versus 50 Placebo: 68 versus 27	All patients were Caucasian	Not mentioned	VitD: 4 versus 96 Placebo:19 versus 81	VitD: 50 versus 50 Placebo: 36.7 versus 63.3	VitD: 97 versus 3 Placebo: 92 versus 8
Mean of Body mass index kg/m^2^	Weekly: 30.5 ± 9.8 Monthly: 32.0 ± 9.5 Placebo: 30.1 ± 6.5	VitD: 26.7 Placebo: 26.4	No mentioned	VitD: 25.9 Placebo: 24.6	VitD: 30.1 ± 8.4 Placebo: 27.6 ± 7.7	VitD: 26.6 ± 6.4 Placebo: 31.3 ± 9.5	VitD: 26.7 ± 7.3 Placebo: 26.8 ± 5.9
Duration of the study	16 weeks	15 weeks	12 months	8 weeks	6 weeks	06 months	39 weeks
Baseline serum vitD in vitD group ng/mL	22	13	12	8.5	14	17	17
Administered dose of vitD	Weekly: 50 000 IU/week /4 weeks Monthly: 50.000 IU/ month/3 months	10 000 IU / week/15 weeks	25 000 IU each 2 weeks for 12 months per os	40 000 IU/ week/8 weeks	200 000 IU/ week/ 3 weeks	50 000 IU/ week/8weeks then 50.000 IU/ Month/4 months	25 000 IU/ week/ 13 weeks Then with dose adjustment
Cumulative dose of vitD	Weekly :200 000 IU Monthly :150 000 IU	150 000 IU	600 000 IU	320 000 IU	600 000 IU	600 000 IU	325 000 IU
Type of administered vitD	Ergocalciferol	Cholecalciferol	Cholecalciferol	Cholecalciferol	Cholecalciferol	Cholecalciferol	Cholecalciferol
Mean of serum vitD After supplementation in vitD group ng/mL	Weekly vitD: 48 Monthly vitD: 36 (*p* < 0.001)	23.6 (*p* < 0.001)	33 (*p* < 0.001)	46 (*p* < 0.001)	52.4 ± 18 (*p* < 0.001)	39 ± 10 (*p* = 0.04)	35.2 ± 12 (*p* < 0.001)
Delta drop in serum vitD	27 ng/mL	10 ng/mL	11 ng/mL	51 ng/mL	38 ng/mL	18 ng/mL	18 ng/mL
Percentage of patients who achieve 25 OHD> 30 ng/mL in vitD group	At the 12th week: Weekly arm: 91% Monthly arm: 65%	Not mentioned	At the 12th month : 71%	At the 8th week : 100%	At the 6th week : 90%	Not mentioned	At the 13th week : 70%
Effect on serum calcium	No difference	No difference	No difference	No difference	No difference	No difference	No difference
Effect on serum phosphorus	No difference	No difference	No difference	No difference	No difference	Has decreased	No difference
Effect on serum PTH	No difference	No difference	Has decreased	No difference	No difference	No difference	No difference
Progression of vascular calcification	Not studied	Not studied	Increased calcification scores in both groups	Not studied	Not studied	Not studied	No difference
Value of serum 1.25 OH_2_D	No difference	Has increased	Not studied	No difference	Has increased	Has increased	Has increased
Other effects observed in vitD group	No effect on : FGF-23 Hospitalization 1-year mortality Blood pressure	No difference according to diabetes and ethnicity	Not studied	No effect on muscle function or biomarkers related to cardiovascular diseases	No difference in albumin, hemoglobin and lipidbiological biomarkers	No effect on test of functional capacity or muscle strength	No effect on bone turnover parameters
Adverse effect in vitD group	Similar between the three groups	Not mentioned	No hypercalcemic episodes	Three hypercalcemic episodes	Not mentioned	No difference in falls and fractures	One hypercalcemic episode
Drugs taken Calcitriol, Cinacalcet, Calcium	Calcitriol	Calcitriol	Phosphate binders	Calcitriol, Cinacalcet, phosphate binders	Calcitriol	Calcitriol, calcium	Calcitriol, cinacalcet

The cumulative doses administered throughout the duration of the study show great variation, from 150 000 to 600 000 IU, as do the modes of administration.

In the prospective study of Zitt et al., native vitamin D substitution adapted to the body weight of patients on CHD and peritoneal dialysis did not show significant difference concerning the increase in calcemia and phosphoremia between the start of the study and the end of 26 weeks of treatment, but the decrease in the value of serum PTH was significant [35]. It should be noted that in this study, certain patients were already receiving calcitriol and cinacalcet, which may largely explain the PTH decrease, even though the two groups were initially similar. In a small clinical trial conducted by Mieczkowski et al. in Poland, the nine patients included in the native vitamin D arm (versus a control group) received low doses, 72 000 IU in 12 months, and the difference between the two groups after a year of treatment was significant concerning levels of 25(OH)D and 1.25(OH)_2_D, without significant impact on calcemia, phosphoremia, or PTH [36]. The main limitations of this study are the small sample and lack of a placebo group.

Bhan et al. conducted one of the largest clinical trials, with 105 patients in three groups (placebo versus vitamin D weekly and vitamin D monthly). It did not show statistically significant differences concerning calcemia, phosphoremia, or PTH, with equal incidence of secondary effects between the three groups [28]. However, the weak results of this trial may be largely explained by the low doses of vitamin D administered over short periods (150 000–200 000 IU in 1–3 months).

Armas et al. conducted the first controlled randomized double-blind trial in 2007 with relatively lower vitamin D doses, i.e(0).150 000 IU for 15 weeks, and noted no significant difference between the two groups concerning serum calcium, phosphorus or PTH, even though the 1.25(OH)_2_D increased in the treated group [29]. In 2009, Delanaye et al. conducted a controlled randomized double-blind trial with high vitamin D doses of 600 000 IU, but administered for 12 months, and noted no significant difference concerning serum calcium and phosphorus [30]. In this trial, the authors found that the delta PTH level significantly decreased in the treated group, but all patients received calcitriol, calcium, and phosphate binders, and the level of 1.25(OH)_2_D was not studied. The availability of 1.25 (OH)_2_D in CHD patients is important because availability in physiologic quantity allows optimization of osseous and extra-osseous effects of vitamin D. Its production in this context remains dependent on extrarenal, or intracellular, production that is itself dependent on intracellular availability of an adequate quantity of 25(OH)D. Only sufficient native vitamin D supplementation allows sufficient production of calcitriol.

Marckman et al., Wass et al., Hewitte et al., and Massard et al. [31–34] noted in their respective clinical trials that vitamin D supplementation was accompanied by a significant increase in calcitriol.

The vitamin D target achieved in the majority of these clinical trials was greater than 30 ng/mL and did not exceed 60 ng/mL, reflecting the safety of both low and relatively high doses of vitamin D over short periods of time or long periods. Admittedly, these clinical trials have not shown any major side effects of vitamin D supplementation, particularly increases in calcium and blood phosphorus, which demonstrates the safe use of native vitamin D in CHD. Thus, the benefit of supplementation appears to be greater than the risk of non-supplementation.

All of these trials are important, but do not provide sufficient and accurate scientific evidence concerning the advantages, consequences and secondary effects of native vitamin D supplementation in CHD. The authors themselves recognize their limitations and recommend larger studies including larger numbers of patients with more rigorous inclusion criteria and standardization of protocols for vitamin D administration (ergocalciferol versus cholecalciferol, doses, duration, season of the study, etc.). It is only the native vitamin D fraction combining D_2_ and D_3_ that is usually measured in clinical trials. An unavoidable question concerns the involvement of each form in phosphocalcic metabolism and the extra-osseous effects on patients in CHD. Only Bhan et al. [28], in their 2009 clinical trial measured native vitamin D as well as its two fractions D_2_ and D_3_ at the start of the study, but end of study values were not reported in the results.

## International recommendations

In light of the results of these clinical trials, what are the recommendations of the main learned societies of nephrology for CHD patients? Table 2 summarizes the main recommendations for native vitamin D supplementation in CHD patients according to the several international societies of nephrology. In 2003, Kidney Disease Outcomes Quality Initiative (KDOQI) recommended a correction of vitamin D to reach a threshold of 30 ng/mL without specifying the stage of CKD [37]. In 2017, KDIGO recommended a correction of vitamin D deficiency and insufficiency for CKD from stages 3a–5 dialysis without specifying the target threshold [38]. In 2003, KDOQI had recommended, but only for CKD stages 3 and 4, a measurement of vitamin D (evidence*) and ergocalciferol (D_2_) supplementation if the 25(OH)D value was <30 ng/mL (opinion*). The recommended dose was 50 000 IU per week or month for 6 months depending on the severity of the deficiency. In these 2003 recommendations, the dialyzed patient in stage 5D was not mentioned and the authors questioned the pertinence of supplementing with vitamin D, given the low renal production of 1.25(OH)_2_D. However, recent discoveries in fundamental and clinical research have shown the persistence of weak renal production and the presence of quite high extrarenal production of 1.25(OH)_2_D, which points anew to the importance of vitamin D supplementation in CHD patients [39].

**Table 2.** The main recommendations concerning native vitamin D supplementation in adults chronic hemodialysis patients.

Guidelines	Recommendations for chronic hemodialysis patients	Thresholds	Dose	Duration	Molecule
The KDIGO 2009 (Kidney Disease Improving Global Outcomes) [4]	In patients with CKD stages 3–5 D, we suggest that 25(OH)D (calcidiol) might be measured, and repeated testing determined by baseline values and therapeutic interventions (2 C). We suggest that vitamin D deficiency and insufficiency be corrected using treatment strategies recommended for the general population (2 C)	None	None	None	None
The KDOQI 2003 (Kidney Disease Outcomes Quality Initiative) [37]	No recommendation about native vitamin D for dialysis patients. In patients with GFR< 20 mL/min/1.73 m^2^ and those requiring dialysis, there is no evidence that modest supplementation with ergocalciferol to raise serum 25(OH)D levels to 30–60 pg/mL will increase the plasma levels of 1,25 (OH)_2_D or lower the elevated serum levels of intact PTH	None	None	None	None
KDIGO 2017 [38]	The same recommendation as KDIGO 2009. In patients with CKD G3a–G5D, we suggest that 25(OH)D levels might be measured, and repeated testing determined by baseline values and therapeutic interventions (2 C). We suggest that vitamin D deficiency and insufficiency be corrected using treatment strategies recommended for the general population (2 C)	None	None	None	None
The KDOQI 2010 Commentary on KDIGO 2009 [40]	Needs to individualize the decision for whether, when, and how often to measure vitamin D and below what threshold and to what target range to treat. A reasonable approach is to periodically measure 25(OH) D in patients with CKD and initiate treatment if the level is low	None	None	None	None
The CSN 2010 Commentary on KDIGO 2009 (Canadian Society of Nephrology) [41]	Until the clinical benefit of correcting nutritional vitamin D ‘insufficiency’ has been established within the dialysis population or for patients with stages 4 and 5 CKD, it would seem premature to suggest screening with vitamin D assays	None	None	None	None
The CARI 2006 (Caring for Australasians with Renal Impairment) [42]	No recommendations about native vitamin D for dialysis patients	None	None	None	None
KWG 2015 (Korean working Group) [43]	None	None	None	None	None
JSDT 2013 (The Japanese Society for Dialysis Therapy) [44]	None	None	None	None	None
The ERBP 2010 Commentary on KDIGO 2009 (European Renal Best Practice) [45]	None	None	None	None	None
UKAR 2011 (United Kingdom Report Annual final version 2015) [46]	Although the benefits are unconfirmed, a reasonable case exists for the measurement of vitamin D in CKD. A pragmatic approach is to measure 25(OH)D at baseline in CKD stage 3 b and above, with a view to correction of insufficiency or deficiency (>75 nmol/L = repletion, 37.5–75 nmol/L = insufficiency, <37.5 nmol/L = deficiency). Vitamin D levels need to be interpreted in the context of the patient's overall clinical condition, other biochemical abnormalities, and the preexisting therapy. Repeat testing will be determined by baseline value and therapeutic interventions	None	None	None	None

In 2009, KDIGO suggested, with a lower level of proof, measuring 25(OH)D serum concentration in CKD patients in stages 3, 4, 5, and 5 dialyzed (2 C), and treating as for the general population, but favoring this time form D_3_ of vitamin D and not the D_2_ form as in 2003 (2 C) [4]. The lack of clear recommendations by KDOQI 2003 and KDIGO 2009 explains the enthusiasm and interest brought by certain authors to carrying out clinical trials, mentioned in Table 1, and which tried to provide clear answers, but without strong scientific proof. In addition, none of the American, Canadian, Australian, Asian, European, and English recommendations have specified the targets, doses, duration or the molecule of vitamin D supplementation in the patient on CHD [40–46]. The European recommendations of 2010 European Renal Best practice (REBP) opted for native vitamin D 12.5 ng/mL as the treatment threshold instead of the threshold of 30 ng/mL, due to the absence of a clear benefit from supplementation beyond 12.5 ng/mL [45]. KDOQI 2003 had recommended the oral use of ergocalciferol (and not cholecalciferol) at relatively high doses of 600 000 IU in 6 months, while ERBP 2010 preferred cholecalciferol without mention of modes of administration. KDIGO 2009 gave no specifications concerning either the molecule or the doses and recommended the same treatment strategy as for the general population.

It is currently well-established that in using spaced doses of vitamin D in the general population, vitamin D_3_ is superior to vitamin D_2_. The half-life of 25(OH)D_3_ is longer than that of 25(OH)D_2_ making it possible to achieve a satisfactory vitamin status for a long time [47]. In 2017, the long awaited KDIGO recommendations were published and despite the results of clinical trials conducted between 2007 and 2013, the recommendations on vitamin D supplementation in CHD were very imprecise and sparse, limited to suggesting correction of any state of insufficiency or deficiency in patients presenting CKD of stage 3A to stage 5D (2C). In this recommendation, CHD patients are considered as patients in CKD stage 3A without taking into account either the special characteristics of CHD patients, or the marked prevalence of vitamin D insufficiency in CHD, or the strong correlation with morbi-mortality in CHD, or the probably greater need for supplementation in order to reduce the harmful consequences of vitamin D insufficiency on phosphocalcic metabolism, the cardiovascular system, immunity and numerous other systems, and functions.

## Conclusion

Many questions remain unanswered for CHD patients, who are at high cardiovascular risk with high morbi-mortality: the precise role of each fraction of vitamin D, ergocalciferol versus cholecalciferol; the isolated impact of each fraction of vitamin D on different functions; the optimal target thresholds and the toxicity threshold of native vitamin D; the dose to be administered and treatment duration. Would it not also be more pertinent to supplement in relation to the weight and lean body mass of the patient rather than with standard doses? Only clinical trials that are larger, more rigorous, and more precise with the fewest possible interferences can provide clear answers and guide working groups to more appropriate recommendations that are better adapted to patients in CHD.
